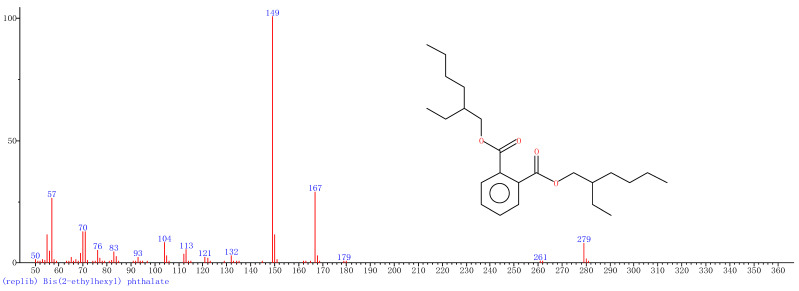# Green Synthesis of Iron Nanoparticles Using Green Tea and Its Removal of Hexavalent Chromium

**DOI:** 10.3390/nano11030650

**Published:** 2021-03-08

**Authors:** Runqin Hao, Dong Li, Jie Zhang, Tifeng Jiao

**Affiliations:** 1 Key Laboratory of Water Quality Science and Water Environment Recovery Engineering, Beijing University of Technology, Beijing 100124, China; hrunqin@163.com (R.H.); 6282031@163.com (J.Z.); 2 Environmental Protection Research Institute of Light Industry, Beijing 100089, China; 3 State Key Laboratory of Urban Water Resource and Environment, Harbin Institute of Technology, Harbin 150090, China; 4 State Key Laboratory of Metastable Materials Science and Technology, Yanshan University, Qinhuangdao 066004, China

**Keywords:** Cr(VI), iron nanoparticles, green tea extract, removal efficiency

## Abstract

Chromium (VI) is a ubiquitous groundwater contaminant and it is dangerous to both ecological and human health. Iron nanoparticles (*n*Fe) have a large specific surface area and they are highly efficient in removing chromium (VI) from aqueous solution. However, since the traditional reductive synthesis of *n*Fe is relatively expensive and often causes secondary pollution, it is necessary to develop a low-cost green synthetic method using plant extracts. Synthetic conditions are important for obtaining highly active chromium-removing nanomaterials. In this paper, a green tea extract was used to prepare *n*Fe and the effects of synthetic conditions on subsequent remediation performance were investigated. The optimal conditions included a green tea extract/Fe^2+^ ratio of 1:2 (91.6%), a green tea extract temperature of 353 K (88.3%) and a synthetic temperature of 298 K (88.1%). Advanced material characterization techniques, including XPS, SEM-EDS, TEM, and Brunauer–Emmett–Teller (BET) confirmed that the average particle size was between 50–80 nm, with a specific surface area of 42.25 m^2^·g^−1^. Furthermore *n*Fe had a core-shell structure, where Fe (0) constituted the core and a shell was composed of iron oxide. Finally, a mechanism for synthesizing *n*Fe by green tea extract was proposed, providing a theoretical basis for optimized synthetic conditions for preparing *n*Fe when using green tea extract.

## 1. Introduction

Many common industrial activities, such as electroplating, printing, dying and leather processing generate large volumes of metal-rich waste streams, which without appropriate treatment can lead to significant contamination of surface water, groundwater and soil. Of the large number of metal contaminants deposited into the environment, chromium (Cr) is one metal that has attracted much concern owing to its high toxicity even at very low concentrations [1,2]. While chromium exists in two major oxidation states, Cr(III) and Cr(VI), Cr(VI) is 500 times more toxic than Cr (III); it is mainly Cr(VI) exposure that is associated with human health risks, as Cr(VI) toxicity may damage human organs including the kidneys and liver, cause dermatitis and trigger gastrointestinal ulcers [1,2]. Therefore, removing chromium from wastewater is urgent. Traditional methods including adsorption, chemical precipitation, electrocoagulation, ion exchange, electrodialysis, and membrane separation are usually used to remove chromium. Adsorption has a high removal efficiency, simple operation and low cost, so it has emerged as the best way to remove chromium from wastewater. Recently, since reducing the levels of toxic substances in aquatic ecosystems and promoting water reuse after treatment has become more popular, calls to use nanomaterials for environmental remediation have gained much momentum [3,4,5]. Until now, various materials have been successfully used for Cr(VI) removal from wastewater including iron nanoparticles [6], graphene oxide [7], carbon nanotubes [8], and metal-organic frameworks(MOFs) [9]. Ling used sodium borohydride synthesized iron nanoparticles to remove Cr(VI) (66%) [6]. Mondal synthesized graphene oxide using Hummer and Offeman’s techniques for removing Cr(VI) under the pH of 4, and documented a removal efficiency of 92.8% [7]. Ahmed et al., using the chemical vapor deposition method synthesized a high purity carbon nanotube to remove Cr(VI) with a sorption capacity of around 333.30mg·g^−1^ [8]. Zhang et al. prepared 2-cationic MOFs, FIR-53 and FIR-54 through the nanoscale route and this reported a removal capacity of 100 mg·g^−1^ for Cr(VI) [9].

One type of nanomaterial commonly proposed for wastewater treatment is iron-based nanoparticles (*n*Fe), because they possess the advantages of higher intrinsic reactivity on their surface sites due to their small particle size, large specific surface area and the presence of zero-valent iron which is often suitable for the reductive dechlorination of organic molecules [10]. Thus, *n*Fe is frequently used for the urgent removal of various organic and inorganic contaminants from surface or ground waters [11]. Existing traditional methods for *n*Fe synthesis include chemical reduction, hydrothermal synthesis and physical vapor deposition, where chemical liquid-phase reduction is the most widely used method. However, synthesis via chemical reduction has a number of major issues including, firstly, toxicity to natural organisms, and the agglomeration of nanoscale materials leads to a greatly reduced effective contact area and deteriorating remediation performance [12]; secondly, the potential for nanoparticles to pose potential environmental hazards due to their higher solubility in water compared to micron-sized particles. Consequently, in an effort to reduce large-scale production costs and reduce the biological toxicity of traditionally produced *n*Fe, alternative methods for the synthesis of environmentally friendly, low-cost, and reliable nano-iron materials have been the subject of much research attention in recent years [13].

During the green synthesis of *n*Fe, simple iron salts are reduced to zero-valent iron by biomolecules contained in natural plant extracts [13,14]. Compared with traditional synthesis technologies, green synthesis avoids the use of innately toxic and hazardous chemicals, reduces energy consumption, and thus has the advantages of being environmentally friendly and more easily dispersed. Several natural plants, including green tea and eucalyptus leaves, have been used for the synthesis of iron-based nanoparticles [13,14], which have thereafter been shown to be ideal for the elimination of dyes, halogenated hydrocarbons and heavy metals [15,16,17]. The advantages of using plant extracts for green synthesized iron nanoparticles include simplicity, high efficiency, and sustainability. Recently, oolong tea extracts were used to synthesize predominantly spherical *n*Fe with diameters ranging from 40 to 50 nm for the degradation of malachite green (MG) dye, removing 75% of an initial MG dose of 50 mg·L^−1^ with pseudo first-order reaction kinetics [15]. In addition, *n*Fe synthesized using three different tea extracts served as a catalyst for the Fenton-like oxidation of monochlorobenzene (MCB), degrading 69%, 53%, and 39% of MCB when using green, oolong, and black tea extracts, respectively. This study indicated that the type of extract had some effect on the resulting *n*Fe degradation efficiency.

Green tea extract synthesized *n*Fe demonstrated the best overall degradation, based on the initial adsorption of MCB to the *n*Fe surface, with the decomposition of H_2_O_2_ resulting in hydroxyl radical generation which consequently resulted in MCB oxidation [12]. While application of zero-valent iron in metal remediation has been less commonly proposed, recently *n*Fe synthesized using an eucalyptus leaf extract showed 100% Cr(VI) removal efficiency at a leaf extract and iron (III) solution ratio of 2:1 (v/v) and pH 4 [13]. In this study, the *n*Fe produced had a diameter of 95 nm with FT-IR indicating a capping layer containing polyphenols and aliphatic acids [11]. Meanwhile, XPS revealed that *n*Fe contained both iron oxides and a covering layer of eucalyptus leaf extract-derived biomolecules. However, despite these successes, some knowledge gaps in *n*Fe synthesized by green tea extract still exist, including how specific synthetic conditions impact on the activity of *n*Fe in general and their efficiency for Cr (V) removal. To address these important discrepancies in our knowledge, this study specifically examined: (1) how the synthetic conditions affected the formation of *n*Fe and their Cr(VI) removal efficiency; (2) the specific biomolecules present in green tea extract and involved in the formation of *n*Fe.

Therefore, the main objectives of this work were to: (1) investigate the optimal conditions for *n*Fe synthesis; (2) characterize the *n*Fe so formed; (3) analyze the main biomolecules in the green tea extract which act as reducing or capping agents; (4) propose a formation mechanism of *n*Fe when using green tea extract.

## 2. Materials and Methods

### 2.1. Materials

Green tea was purchased from local tea factories in Hangzhou, Zhejiang Province. FeSO_4_·7H_2_O was supplied by Sinopac Chemical Reagent Co. Ltd. (Danyang, Jiangsu, China). Commercial *n*Fe was provided by Beijing Deke Daojin Science and Technology Co., Ltd. (Beijing, China). The sample was synthesized by sodium borohydride, and its specific surface area was 20 m^2^·g^−1^ and average size amounted to 50 nm. All chemical reagents were analytically pure.

### 2.2. Synthesis of nFe

Prior to the iron nanoparticle (*n*Fe) being synthesized, the purchased green tea was washed liberally with distilled water and dried for 12 h at 333 K. Thereafter, an aliquot of the dried green tea (40 g) and distilled water (1 L) was placed in a 1 L conical flask and heated in a water bath for 60 min at four different temperatures (313, 333, 353, and 373 K), prior to cooling to room temperature and filtering through 0.45 μm disposable filters to obtain a 40 g·L^−1^ green tea extract (GT). An iron slat solution was prepared by dissolving FeSO_4_·7H_2_O (27.8 g) in distilled water (1 L). Thereafter, aliquots of this solution were mixed with GT (30 mL) at five different volume ratios (1:3, 1:2, 1:1, 2:1, and 3:1) in a 150 mL conical flask, where the synthesized *n*Fe was facilitated by heating it in a constant temperature oscillating chamber (298, 308, 318 or 318 K, 150 rpm) for 60 min. Then, the *n*Fe sample was washed three times using anhydrous ethanol and deionized water, respectively.

### 2.3. Characterization

Surface micromorphology and structure of the green tea extract-derived *n*Fe was analyzed using Merlin Compact scanning electron microscopy (SEM) (Zeiss, Baden-Wurttemberg, Germany) and transmission electron microscopy (TEM) (Micrometer, JEOL, Beijing, China). To determine functional groups on the *n*Fe surface the analysis was done employing Fourier transform infrared spectra (FT-IR) (Nicolet IS 50, Thermo Fisher, Waltham, Massachusetts, USA) and the recording range was set at 400 to 4000 cm^−1^. Surface chemical compositions of *n*Fe were analyzed using X-ray photoelectron spectroscopy (XPS) (AXIS Supra, Kratos Analytical, Trafford Park, Manchester, UK). Meanwhile the surface area and nanoparticle size were analyzed using N_2_-Brunauer–Emmett–Teller isotherm (BET) (ASAP 2020 Plus HD88, Shanghai, China).

The composition of the green tea extract before and after *n*Fe synthesis was analyzed using a gas chromatography-mass spectrometer (GC-MS) on a 7890B Agilent GC instrument equipped with a mass selective detector (MSD) (HP 5977C) quadrupole MS (Palo Alto, Agilent Technologies, Palo Alto, California, USA). Helium (99.999%) at a flow rate of 1.0 mL·min^−1^ served as the carrier gas while chromatographic separation was conducted through a HP-5MS (30 μm × 250 μm × 0.25 μm) capillary column. Samples were introduced into the GC-MS system in splitless mode and the injector and ion source temperatures were set to 280 and 230 °C, respectively. The GC oven temperature was initially maintained at 50 °C for 2 min, prior to being increased from 50 to 280 °C at a heating rate of 6 °C·min^−1^. The GC-MS operating mode was set to use 70 eV of ionization energy for electron bombardment, and the temperatures of the quadrupole and ionization source were set to 150 and 250 °C, respectively. The mass spectra were obtained for the m/z ratio in a full scan range with a 5.5 min solvent delay.

### 2.4. Removal of Cr(VI) by nFe

The removal efficiency of *n*Fe was investigated using a batch process. This study used the *n*Fe synthesized under optimal conditions. These conditions included a GT concentration of 40 g·L^−1^, a GT:Fe^2+^ ratio of 1:2, a GT extract temperature of 353 K and a synthesis room temperature of 298 K. During batch studies, a specific dose of *n*Fe was exposed to an 80 mg·L^−1^ solution of Cr(VI) (50 mL) for predefined times (2, 4, 6, 8, 10, and 12 h) at 298 K and 150 rpm in an incubator. At each sampling time, an aliquot of the supernatant reaction solution was removed and filtered < 0.45 μm to measure the absorbance of Cr(VI) at 540 nm using an ultraviolet and visible spectrophotometer (UV-vis) (UV3600PLUS, Shimadzu).

## 3. Results and Discussion

### 3.1. Effect of Synthesizing Conditions on Removal Efficiency

**Figure 1:**
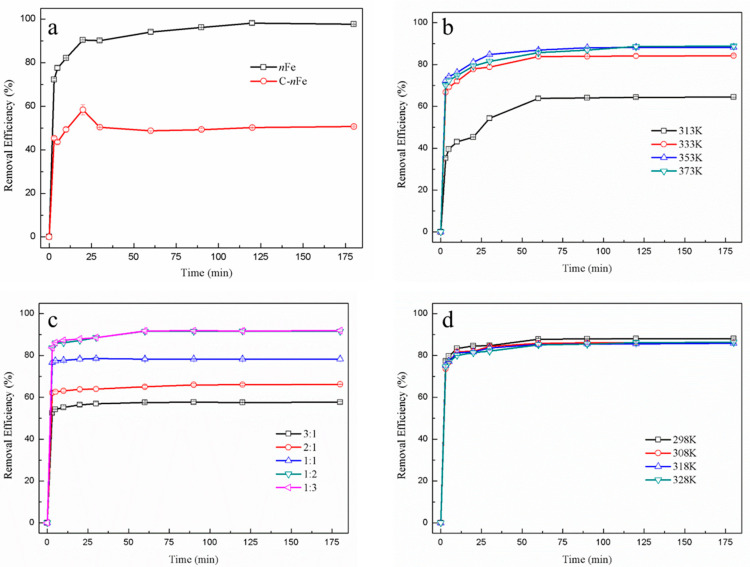
Temporal variation in Cr(VI) removal efficiency for green synthesized *n*Fe (black line) and commercially purchased *n*Fe (C-*n*Fe) (red line) (**a**) with different synthesis conditions, including green tea extract water bath temperature (313, 333, 353, and 373 K) (**b**); the ratio of GT:Fe^2+^ (3:1, 2:1, 1:1, 1:2, and 1:3) (**c**); synthesis temperature (298, 308, 318, and 328 K) (**d**).

In order to obtain superior functional *n*Fe, various synthesis conditions involving GT bath temperature, synthesis temperature and Fe^2+^ to green tea extract ratio of *n*Fe were controlled to make possible the removal of hexavalent chromium (Figure 1). The absorbance of Cr(VI) was measured using an UV-vis in this paper, which mainly focused on the removal of Cr(VI).

The removal efficiency of Cr(VI) using green synthesized *n*Fe produced here (97.66%) was much higher than that of commercially sourced *n*Fe (50.68%) at 180 min (Figure 1a). This improvement in Cr(VI) removal efficiency when utilizing green synthesized material might be attributed to the presence of biomolecules on the *n*Fe surface, which contain a myriad of carboxyl, hydroxyl and other functional groups. They can combine Cr(VI) to form a covalent bond, resulting in much better removal efficiency. On the other hand, biomolecules on the *n*Fe surface can reduce the agglomeration of *n*Fe to enhance efficiency in Cr(VI) removal. Irrespective of the final *n*Fe sources, the time to reach equilibrium for both was about 30 min, suggesting that the iron particles synthesized by green tea had the same overall function as that of the commercially available sample. Nonetheless they proved to be more efficient in removal capacity.

While the Cr(VI) removal efficiency tended to increase when the green tea extract temperature also rose (Figure 1b) and reached its highest (88.25%) at 353 K, the removal efficiency was not significantly higher for the three temperatures above 313 K (333, 353, and 373 K). This suggested that some biomolecules in green tea important for Cr(VI) removal were not efficiently extracted when the water batch temperature was <333 K, potentially resulting in less functionalized *n*Fe [18,19].

The ratio of GT:Fe^2+^ used in the synthesis of *n*Fe affected the Cr(VI) removal efficiency (Figure 1c). For ratios of GT:Fe^2+^ as low as 1:2, the removal efficiency remained unchanged. However, as the GT:Fe^2+^ ratio increased from 1:2 to 1:3, there was a subsequently slight increase in the removal efficiency from 91.6% and 91.9%, respectively. These results generally showed that Cr(VI) removal efficiency increased with Fe^2+^, indicating that the amount of Fe in the *n*Fe played an important role in Cr(VI) removal [20,21]. Besides, the overall difference in removal efficiency was minor, because the biological molecular weight of green tea extract may not be enough to synthesize iron nanoparticles from excess iron divalent ions [22].

The removal efficiency of Cr(VI) did not significantly alter when synthesis temperature varied between 298 and 328 K (Figure 1d). Consequently, temperature did not affect *n*Fe activity. Considering the large-scale economics of production, the best scenario for *n*Fe synthesis would be simple room temperature.

During synthesis, the best Cr(VI) removal was obtained at a GT:Fe^2+^ ratio of 1:2, at a temperature to leach GT of 353 K and a temperature to synthesize *n*Fe at room temperature. The removal efficiency of green synthesized *n*Fe for hexavalent chromium was much higher than that of commercially purchased *n*Fe, which suggests that green tea molecules may wrap around the *n*Fe, while the functional groups of green tea molecules could form chemical bonds with hexavalent chromium for removal. Whether Cr(VI) could be reduced to Cr(III) by *n*Fe and remain in solution was not clear. Atomic emission spectroscopy can detect the valence and concentration of both Cr(VI) and Cr(III), which has been documented in one study [23]. The structure and morphology of *n*Fe prepared under these optimal conditions were subsequently examined to understand the process.

### 3.2. Nanoparticle Characterization

**Figure 2:**
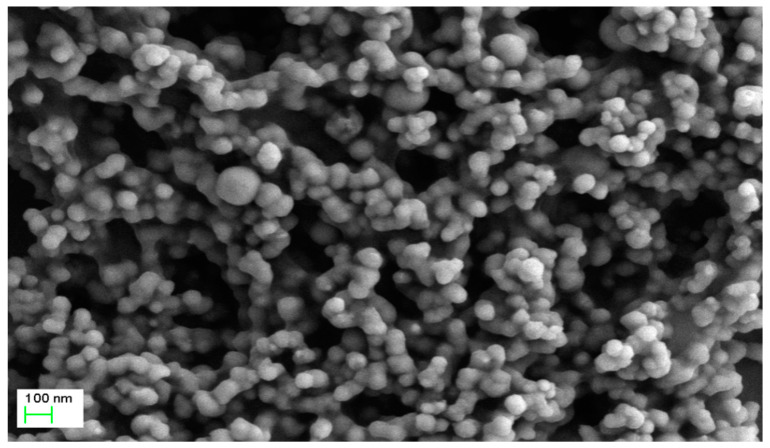
SEM image of *n*Fe prepared using green tea extract. Conditions: accelerating voltage of 3 kV and indicated magnification of 20k×.

**Figure 3:**
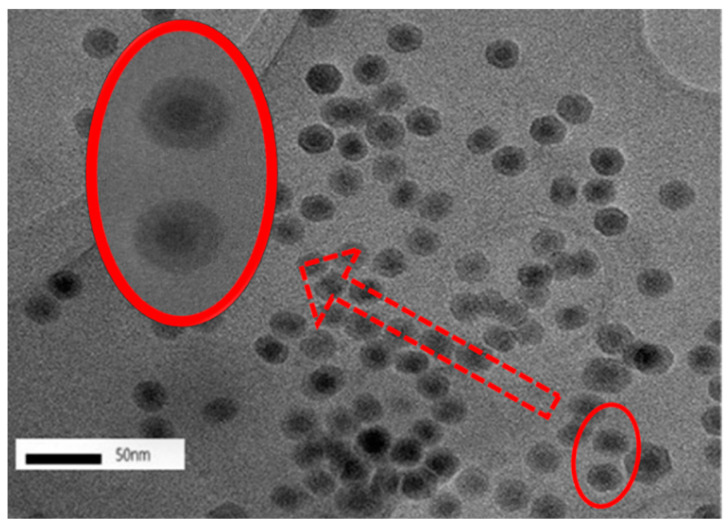
TEM images of *n*Fe. Conditions: accelerating voltage of 200 kV, indicated magnification of 100k×.

Consistent with previous studies [24,25], SEM revealed that the produced *n*Fe were uniformly dispersed, spherical in shape and with an average diameter of 50–80 nm (Figure 2). A few agglomerated particles were more than 100 nm in size with a chain-like structure, mainly due to the strong magnetism and van der Waals attraction between *n*Fe [26,27]. It was thus concluded that the use of GT during *n*Fe synthesis is beneficial, since GT acted as a dispersant during synthesis and reduced agglomeration. In order to further understand the internal structure and morphology of *n*Fe, TEM characterization was performed below (Figure 3).

Under magnification, the TEM images indicated that the *n*Fe exhibited a core-shell structure (Figure 3). The core-shell structure has been reported previously [28], where zero-valent iron nanoparticles constitute a core surrounded by a layer of oxidized iron. It has been reported that the *n*Fe was well dispersed with little agglomeration, which was attributed to the organic compounds derived from the bioactive substances in the green tea extract. This acted as a protective dispersion and stabilizer, reducing both oxidization and agglomeration [17]. Furthermore, the dynamic light scattering (DLS) helped to confirm “non clustering” according to one report [14]. While the observance of a core-shell structure is fairly common, using green tea extract to coat the formed *n*Fe with biomolecules might contribute to some differences from the chemically synthesized *n*Fe [29,30]. To further understand the valence state of the central iron nanoparticles and the nature of the GT coating, XPS characterization was carried out.

**Figure 4:**
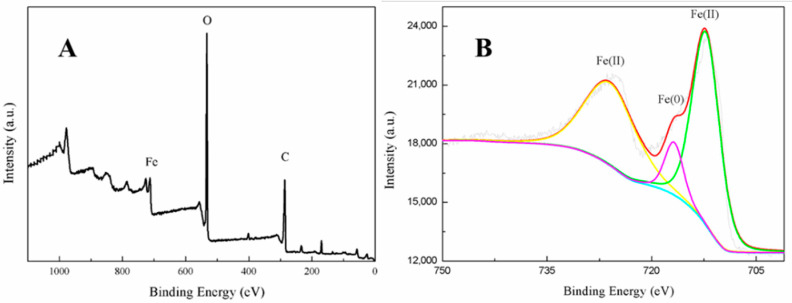
XPS images of *n*Fe, full scan image (**A**) and expanded Fe region (**B**).

The full range XPS scan image of the fabricated nanoparticles indicated they contained three main elements, Fe, C, and O (Figure 4A), confirming that biomolecules derived from the green tea extract were located on the iron nanoparticles’ surfaces [17]. This was consistent with the TEM analysis. Agreeing with previous studies [31,32], peak fitting of the iron region (Figure 4B) indicated that the valence of the iron was distributed between Fe (II) and Fe (0). This further confirmed that zero-valent iron nanoparticles were likely to be present in the core, proving that the green tea extract had reduced Fe^2+^ to Fe (0) and thus acted as a reducing agent. It was, however, surrounded by a layer of ferrous oxide nanoparticles forming a core-shell structure. In order to determine the adsorption behavior and specific surface area of *n*Fe, BET characterization was carried out as follows.

**Figure 5:**
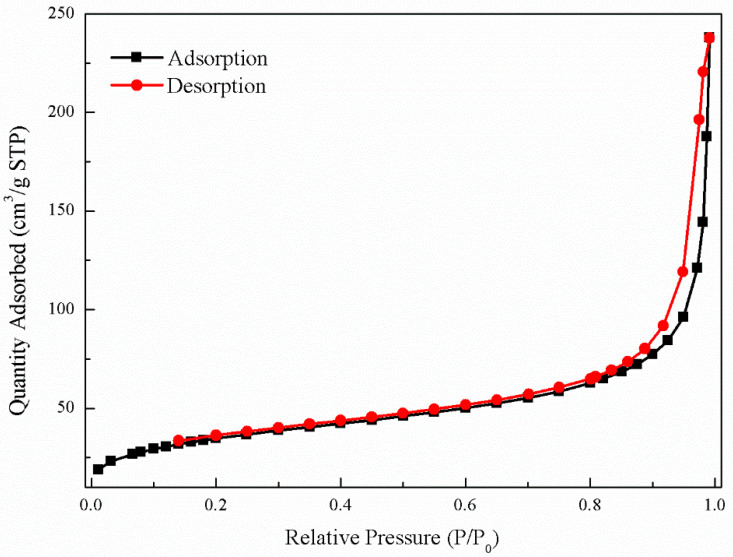
BET isotherm of *n*Fe.

The N_2_ Brunauer–Emmett–Teller isotherm (BET) of *n*Fe (Figure 5) exhibited a type IV nitrogen adsorption-desorption isotherm character, indicative of multi-layer adsorption. The adsorption-desorption hysteresis loop was classified as H3, suggesting that the *n*Fe might contain slit-like pores [33]. The specific surface area of *n*Fe was 42.25 m^2^·g^−1^, which was larger than that of commercially purchased *n*Fe (20 m^2^·g^−1^), meaning that the overall *n*Fe synthesized by GT had a large specific surface area. This was attributed to the organic compounds derived from the bioactive substances in the GT acting as a protective dispersion and stabilizer. It could therefore be concluded that SEM, TEM and the large specific area probably contributed to the good efficiency in removing Cr(VI) [17].

### 3.3. Analysis of the Green Tea Extract

In order to further determine the contribution of green tea to the core-shell structure of the iron nanoparticles, FT-IR and GC-MS characterizations were reported in this section.

**Figure 6:**
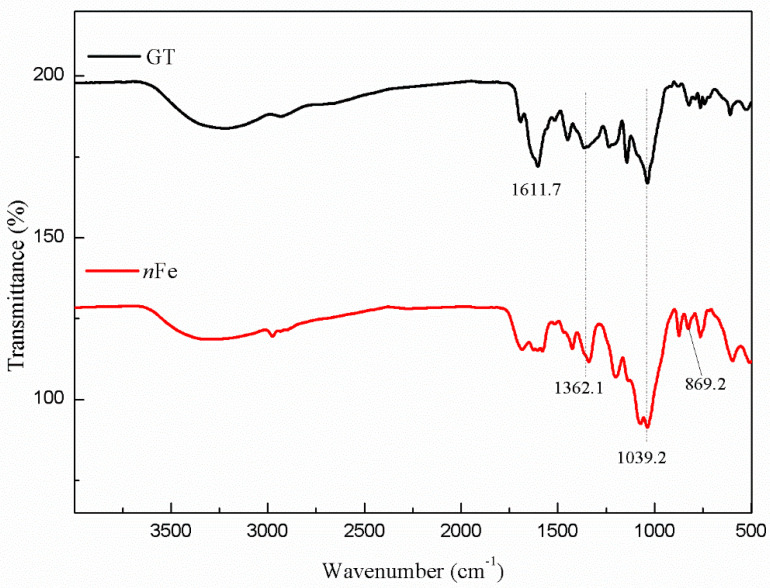
FT-IR spectra of GT (black line) and *n*Fe (red line).

Comparison of the FT-IR spectra of GT and *n*Fe (Figure 6) showed that the peak at 1611.7 cm^−1^ initially present in the green tea extract disappeared after the *n*Fe was synthesized. This peak was attributed to C=C stretching vibrations [34], and its disappearance indicated that biomolecules containing C=C bonds were involved in the synthesis of *n*Fe. Peaks at 1362 and 1039 cm^−1^ in the GT extract, attributable to C-N and C-O-C, respectively, remained largely unchanged following synthesis. It suggests that biomolecules in green tea played a role in the reductive synthesis of *n*Fe, as well as capping agents. Additionally, in the fabricated *n*Fe a new peak appeared at 869.2 cm^−1^, one that was not present in the green tea extract which was ascribed to Fe-O stretching in FeOOH [35]. This scenario is consistent with that of XPS and confirmed that the nanometer iron oxide in the shell was FeOOH. Overall, the FT-IR results were consistent with those from XPS and further supported the presence of iron oxides, and that, while biomolecules containing C=C played a role in the iron reduction, other biomolecules containing C-N and C-O-C acted more as capping agents. Subsequently, GC-MS analysis of the extracts before and after synthesis were carried out to specifically identify which biomolecules were likely to be involved.

**Figure 7:**
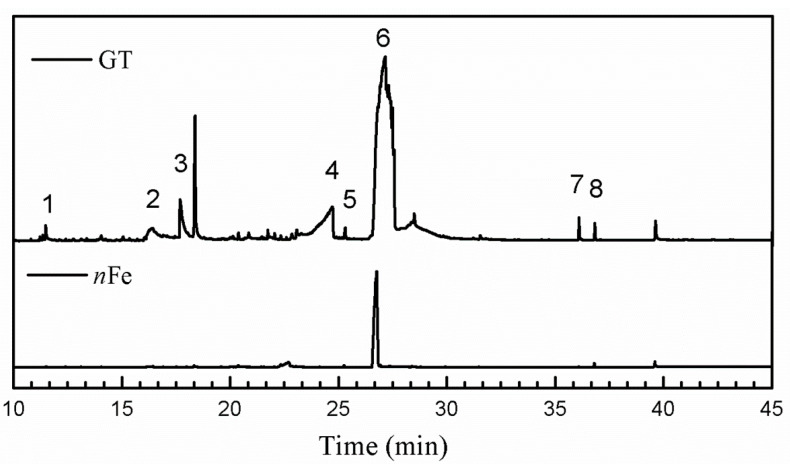
GC-MS chromatograms of GT (upper line) and *n*Fe (lower line).

Comparison of the GT extract before and after the *n*Fe synthesis (Figure 7) confirmed that of the eight peaks present in the original GT extract, seven significant peaks completely disappeared (1, 2, 3, 4, 5, 7, and 8) and one peak significantly decreased (6) following *n*Fe synthesis. This indicated that all of these biomolecules were significantly involved in *n*Fe synthesis. Moreover, since no new peaks appeared after synthesizing *n*Fe, this suggested that the biomolecules originally present were not transformed during synthesis. The specific biomolecules present were identified from the examination of their mass fragmentation pattern and comparison to the MS library match (Table 1). The main constituents of the GT were therefore phenol (11.47 min), 1,1′-Biphenyl, 2-ethyl (16.37 min), 1,2,3-Benzenetriol (17.71 min), 1,3,5-Benzenetriol (24.70 min), 6-Hydroxy-4,4,7a-trimethyl-5,6,7a-tetrahydrobenzofuran (25.33 min), caffeine (27.14 min), oleanitrile (36.09 min), and bis (2-ethylhexyl) phthalate (36.80 min). These results were consistent with those of FT-IR and several previous studies which demonstrated that such GT biomolecules acted both as reducing and capping agents [17].

### 3.4. Mechanism

**Figure 8:**
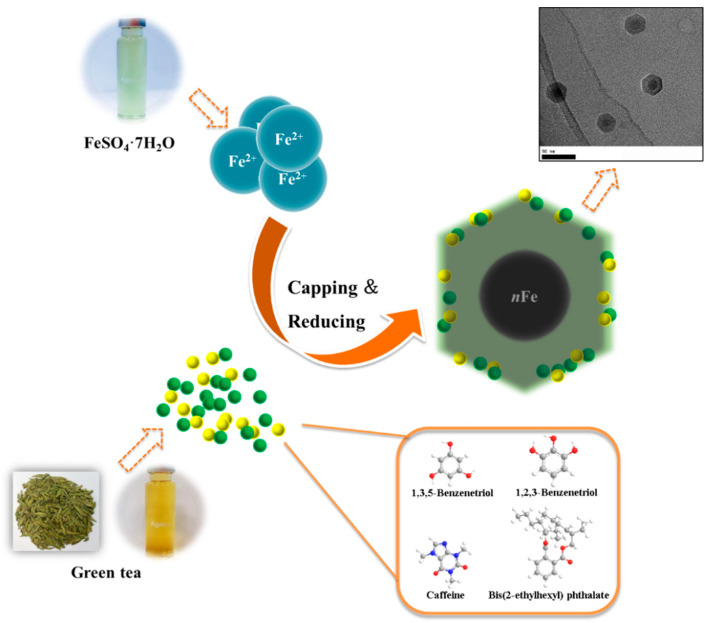
Proposed mechanism of the green tea synthesis of *n*Fe.

When considering all the results holistically, this makes it possible to propose a mechanism for synthesizing green tea extract-derived *n*Fe (Figure 8). Initially, biomolecules present in green tea, such as 1,3,5-Benzenetriol, 1,2,3-Benzenetriol, caffeine and bis(2-ethylhexyl) phthalate, were extracted into an aqueous solution, which, upon mixing with an iron salt, spontaneously led to the formation of core-shell structured *n*Fe. It comprised an Fe (0) core and FeOOH shell, where all of the major biomolecules in the green tea extract acted as the reducing and capping agents, functioning to reduce Fe^2+^ to Fe (0) and cap around the iron nanoparticles.

### 3.5. Stability of nFe Removal Efficiency

**Figure 9:**
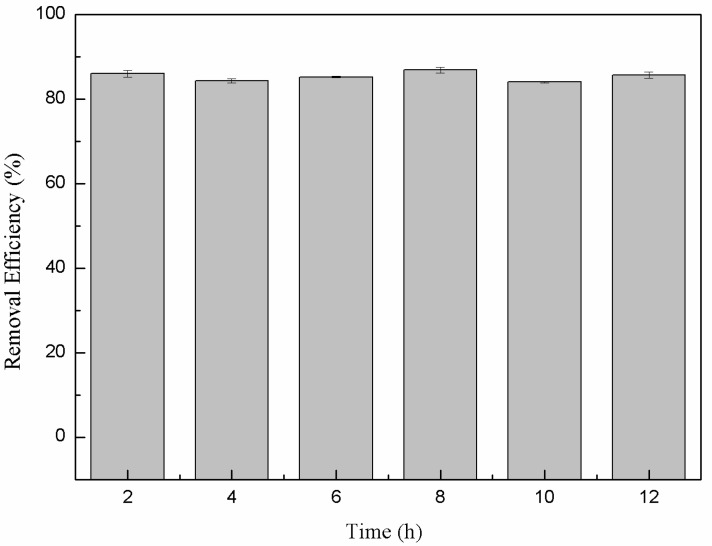
Variation in the removal efficiency of *n*Fe over time.

The long-term stability of the produced nanoparticles in terms of the sustained removal efficiency when exposed to air is an important consideration for long-term practical water treatment. In many cases *n*Fe activity can be significantly reduced due to passivation via iron-oxide surface formation when exposed to air. Here, the Cr(VI) removal efficiency when continually exposed to air remained stable and high (around 85%) for up to 12 h (Figure 9). This indicated that the material retained high stability, which was attributed to the surface coating of bioorganic molecules derived from the green tea extract. They completely encapsulated the iron nanoparticles and prevented oxidation and consequential inactivation. This outcome highlighted that green synthesized *n*Fe has significant practical potential for water treatment strategies.

## 4. Conclusions

A green tea extract was successfully used for the highly efficient (91.6%) green synthesis of *n*Fe to remove Cr(VI) from aqueous solutions. Control of synthesis conditions made it possible to optimize the functional removal efficiency of the nanomaterial produced. SEM and TEM confirmed that the material was a spherical particle with a core-shell structure. XPS showed that the zero-valent iron nanoparticles acted as the core, while iron oxide nanoparticles formed the shell; FT-IR confirmed that the iron oxide shell was FeOOH. GC-MS suggested that most of the biomolecules present in the green tea extract, such as 1,2,3-Benzenetriol, 1,3,5-Benzenetriol, caffeine and bis(2-ethylhexyl) phthalate, were actively involved in the *n*Fe synthesis as reducing or capping agents, which provided a theoretical basis for the green synthesis of nanomaterials. These biomolecules reduced Fe^2+^ to Fe (0) and capped the *n*Fe surface to prevent oxidation and inactivation. This capping led to a high atmospheric stability so that the material could be practically applied more easily than existing materials for water treatment. However, the exact mechanism for removing Cr(VI) by green synthesized *n*Fe is still unclear. Whether green tea also plays a role in this process needs to be analyzed and validated in our next study.

## Figures and Tables

**Table 1:** Mass spectrometry classification of the main biomolecules in GT.

**Entry**	**RT (min)**	**The Major Compound Involved Synthesis**	**Structure**
**1**	**11.47**	**Phenol**	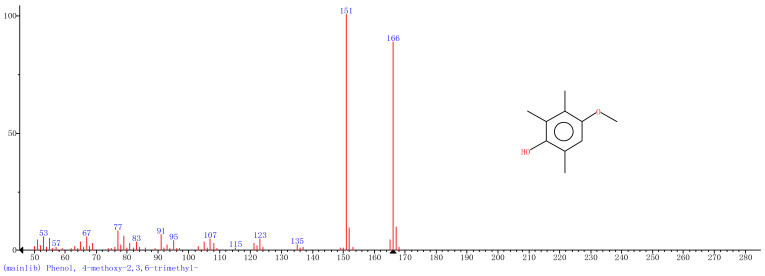
**2**	**16.37**	**1,1′-Biphenyl,2-ethyl-**	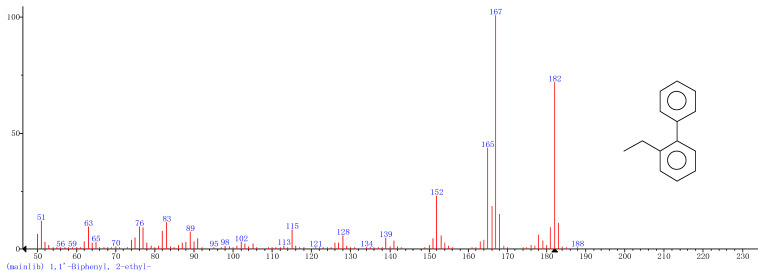
**3**	**17.71**	**1,2,3-Benzenetriol**	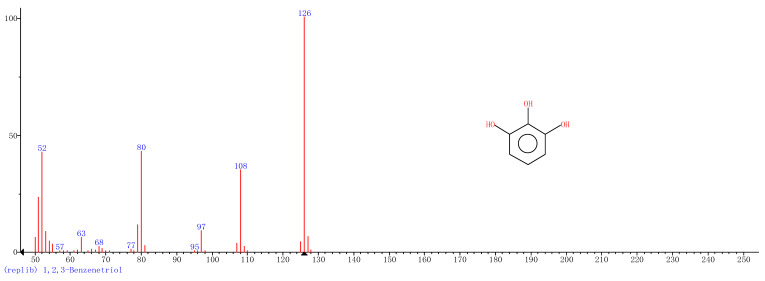
**4**	**24.70**	**1,3,5-Benzenetriol**	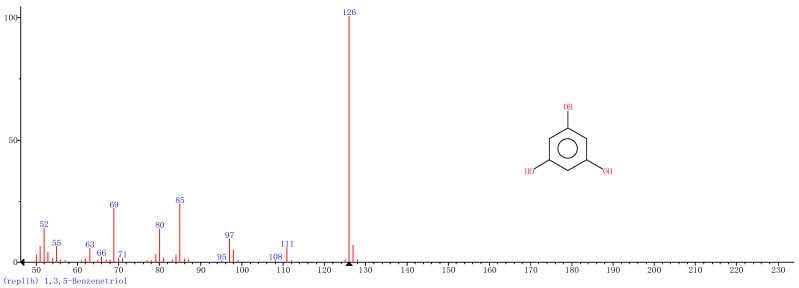
**5**	**25.33**	**6-Hydroxy-4,4,7a-trimethyl-5,6,7a-tetrahydrobenzofuran**	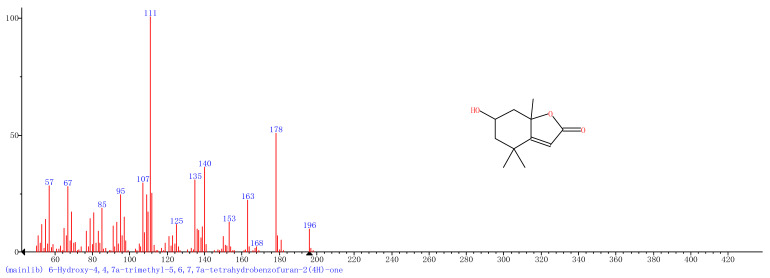
**6**	**27.14**	**Caffeine**	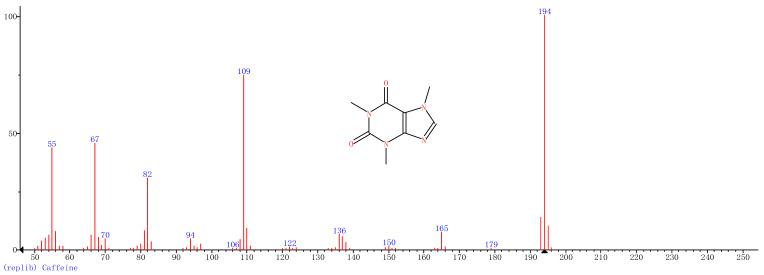
**7**	**36.09**	**Oleanitrile**	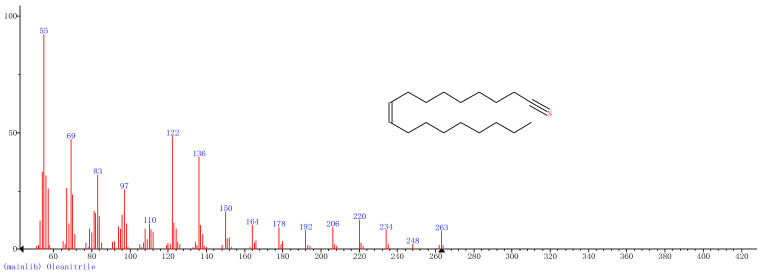
**8**	**36.80**	**Bis(2-ethylhexyl) phthalate**